# Real‐world smartphone data can trace the behavioural impact of epilepsy: A case study

**DOI:** 10.1111/ene.16433

**Published:** 2024-08-07

**Authors:** Arthur R. van Nieuw Amerongen, Anne Marthe Meppelink, Arko Ghosh, Roland D. Thijs

**Affiliations:** ^1^ Stichting Epilepsie Instellingen Nederland (SEIN) Heemstede The Netherlands; ^2^ Department of Neurology Leiden University Medical Center Leiden The Netherlands; ^3^ Cognitive Psychology Unit, Institute of Psychology Leiden University Leiden The Netherlands; ^4^ UCL Queen Square Institute of Neurology London UK

**Keywords:** drug side effects, epilepsy, health technology, neurobehavioral manifestations, smartphone

## Abstract

**Background:**

Neurobehavioural comorbidities have a detrimental effect on the quality of life of people with epilepsy, yet tracking their impact is challenging as behaviour may vary with seizures and anti‐seizure medication (ASM) side effects. Smartphones have the potential to monitor day‐to‐day neurobehavioural patterns objectively. We present the case of a man in his late twenties with drug‐resistant focal epilepsy in whom we ascertained the effects of ASM withdrawal and a convulsive seizure on his touchscreen interactions.

**Methods:**

Using a dedicated app, we recorded over 185 days the timestamps of 718,357 interactions. We divided the various smartphone behaviours according to the next‐interval dynamics of the interactions by using a joint interval distribution (JID). During two ASM load transitions, namely before versus during tapering and tapering versus restarting medication, we used cluster‐based permutation tests to compare the JIDs. We also compared the JID of the seizure day to the average of the previous 3 days.

**Results:**

The cluster‐based permutation tests revealed significant differences, with accelerated next‐interval dynamics during tapering and a reversal upon medication restart. The day of the convulsion exhibited a marked slowing of next‐interval dynamics compared to the preceding 3 days.

**Conclusion:**

Our findings suggest that the temporal dynamics of smartphone touchscreen interactions may help monitor neurobehavioural comorbidities in neurological care.

## INTRODUCTION

Neurobehavioural comorbidities have a detrimental effect on the quality of life in people with epilepsy [1, 2]. Their impact on day‐to‐day behaviour is, however, difficult to monitor as the burden may fluctuate over time due to medication changes or seizure occurrence. The usual approach to quantifying these impairments is to use standardised tests and questionnaires in a hospital‐based setting, thus only providing a snapshot in time.

Smartphones have the potential to monitor day‐to‐day neurobehavioral patterns objectively. Recent work has demonstrated the value of smartphones in various neurological conditions including Parkinson's disease [3]. We demonstrated that smartphone touchscreen interactions can reveal behavioural changes in stroke and epilepsy that are normally associated with ageing [4]. Interestingly, comparing smartphone‐based age estimates with chronological age demonstrated a 10‐year age gap in people with epilepsy, which parallels observations in magnetic resonance imaging (MRI) studies [5]. We postulated that apart from structural brain abnormalities, drug‐induced central nervous system side effects and seizures contribute to the behavioural changes.

We present a single case study of a man in his late twenties in whom we examined the effect of anti‐seizure medication (ASM) changes and seizures on smartphone touchscreen interactions. He participated in a prospective cohort study relating smartphone interactions to self‐reported seizures for 6 months (NCT04617418). During the study, he was admitted to an epilepsy monitoring unit (EMU), for a 5‐day pre‐surgical evaluation. He had drug‐resistant right temporal lobe epilepsy with weekly focal impaired awareness seizures and yearly focal to bilateral tonic–clonic convulsions. No aetiology was identified despite having had several imaging examinations including 3 T and 7 T MRI scans. Positron emission tomography (PET) imaging showed two areas of hypometabolism in the right superior temporal gyrus, while an Ictal single‐photon emission computed tomography (SPECT) scan remained inconclusive. Neuropsychological testing revealed below‐average working memory, but no processing speed, concentration, or executive function problems.

In this case, we leverage the ground truth information on seizures provided by the video‐electroencephalogram (EEG) recordings and the ASM withdrawal linked to the admission.

## METHODS

The Leiden‐Den Haag‐Delft Medical Ethics Committee approved the study, and the subject consented to participate. He installed the TapCounter app (QuantActions, Lausanne) on his smartphone, which recorded the timestamps of all touchscreen interactions, and kept a seizure diary for 6 months. The data from the app automatically transferred to a cloud‐based data collection manager (taps.ai, QuantActions).

**FIGURE 1:**
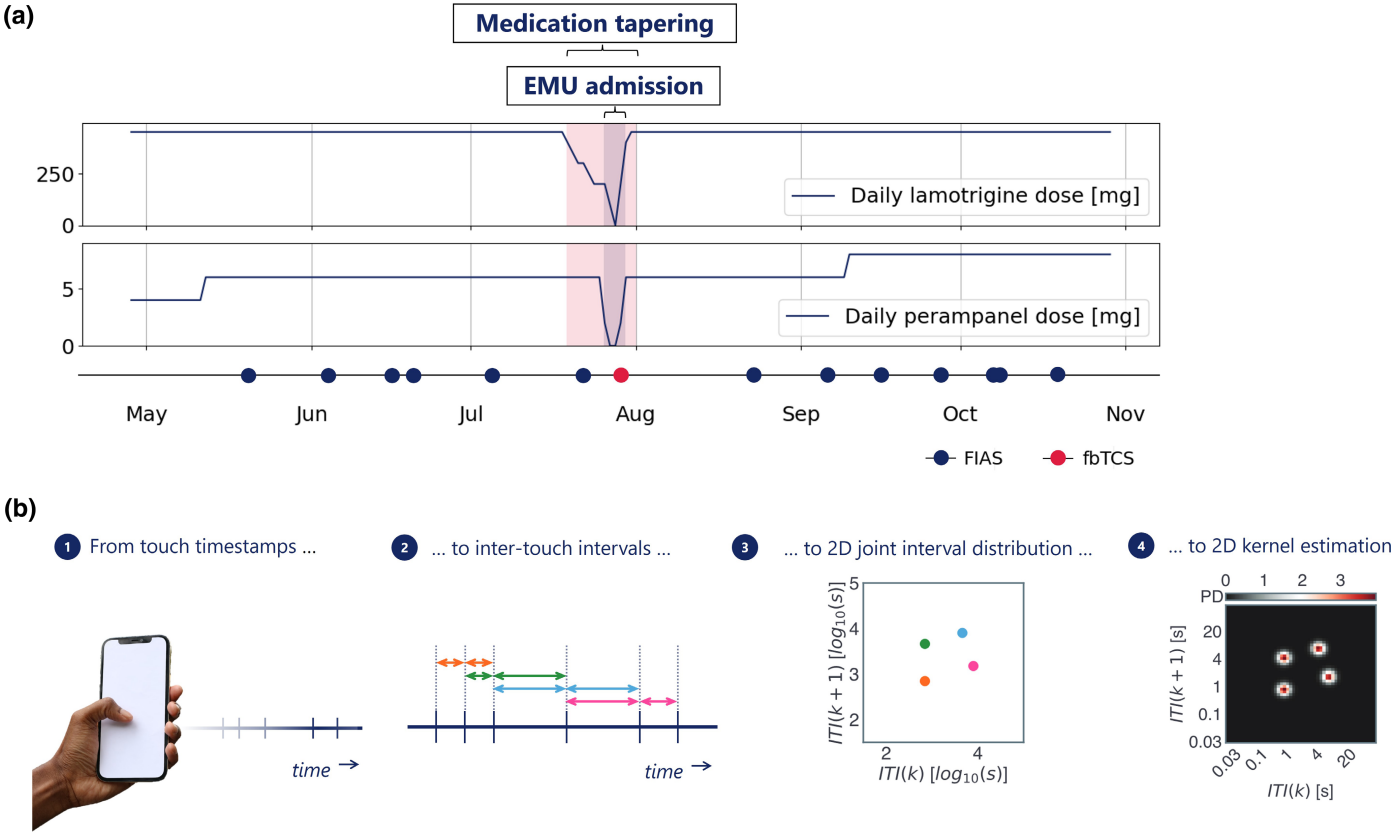
(a) Timeline of the study period. The subject participated from May to November 2021. He reported several seizures, including one convulsion during admission. The anti‐seizure medication load was stable for the study period; his medication was tapered before admission. (b) A smartphone app recorded the timestamps of all touchscreen interactions (1). We calculated the inter‐touch intervals (ITI) (2) and plotted each ITI at time *k* against the following ITI at time *k* + 1 (3). We then estimated a 2D kernel density (4). 2D, two‐dimensional; EMU, epilepsy monitoring unit; fbTCS, focal to bilateral tonic–clonic seizure; FIAS, focal impaired awareness seizure; PD, probability density.

The subject participated from May to November 2021. A total of 718,357 touchscreen interactions were recorded over 185 consecutive days, with a median of 3311 daily interactions (interquartile range [IQR] 2531–4766 interactions). The subject used lamotrigine and perampanel, which were reduced before admission and completely withdrawn during admission (Figure 1a). He reported a total of 14 focal impaired awareness seizures outside the admission period. The 5‐day video‐EEG recording documented nine focal aware seizures with autonomic onset on the first day, four on the second day, one electrographic seizure whilst asleep on each of the first two nights, and one focal to bilateral tonic–clonic convulsion originating from the temporal lobe soon after wake‐up on the fourth day.

We downloaded the data from the online data collection manager. We applied a previously validated sleep and rise time detection algorithm to separate the touchscreen interactions per study day [6]. We then calculated joint‐interval distributions (JIDs) for the inter‐touch intervals (ITIs) from each day (Figure 1b). We created a two‐dimensional (2D) space by relating each ITI (at time *k*) against its subsequent interval (at time *k +* 1). This provides an interpretable separation of behaviors. For example, the lower left corner represents fast interactions and the diagonal represents rhythmic activity. We operated 2D kernel density estimation (Gaussian kernel, bandwidth 0.1) over the log_10_‐transformed 2D space. We subsequently discretised the continuous output using 50 bins per dimension from approximately 30 ms to 100 s, thus obtaining a 50‐by‐50 matrix of 2D bins [7].

To evaluate the impact of ASM tapering on touchscreen interactions we categorised the data into three distinct periods: days before the ASM tapering (*n* = 82), the tapering period itself (12 days) and days after ASM tapering (*n* = 91). We evaluated the contrasts between the daily JIDs from the pre‐tapering period versus the tapering period, and the daily JIDs from the tapering period versus the post‐tapering period.

We employed a permutation‐based clustering approach to identify clusters of 2D bins with significant differences in touchscreen interactions and to account for multiple testing associated with many individual *t*‐tests when comparing JIDs bin‐wise [8]. Specifically, we performed 1000 random label permutations for each comparison (pre‐tapering vs. tapering and tapering vs. post‐tapering). At each iteration, we did a *t*‐test for all 2D bins and identified clusters of contiguous bins with a *t*‐statistic corresponding to *p* < 0.01. This method provided us with a reference distribution of maximal cluster sizes under the null hypothesis that both groups are indistinguishable. This allowed us to assess the significance of cluster sizes observed in our original data. We considered a cluster size above the 95th percentile of the reference distribution as statistically significant.

To assess the seizure effect, we compared the day of the convulsion during EMU admission (Day 4) with the previous three days. We first isolated the touchscreen interactions on the day of the convulsion and computed a JID for that day. Notably, this convulsion‐related JID contains mostly post‐ictal data, as the convulsion occurred shortly after awakening. We then computed the average JID from the 3 days before the convulsion and contrasted it with the JID of the convulsion day.

## RESULTS

**FIGURE 2:**
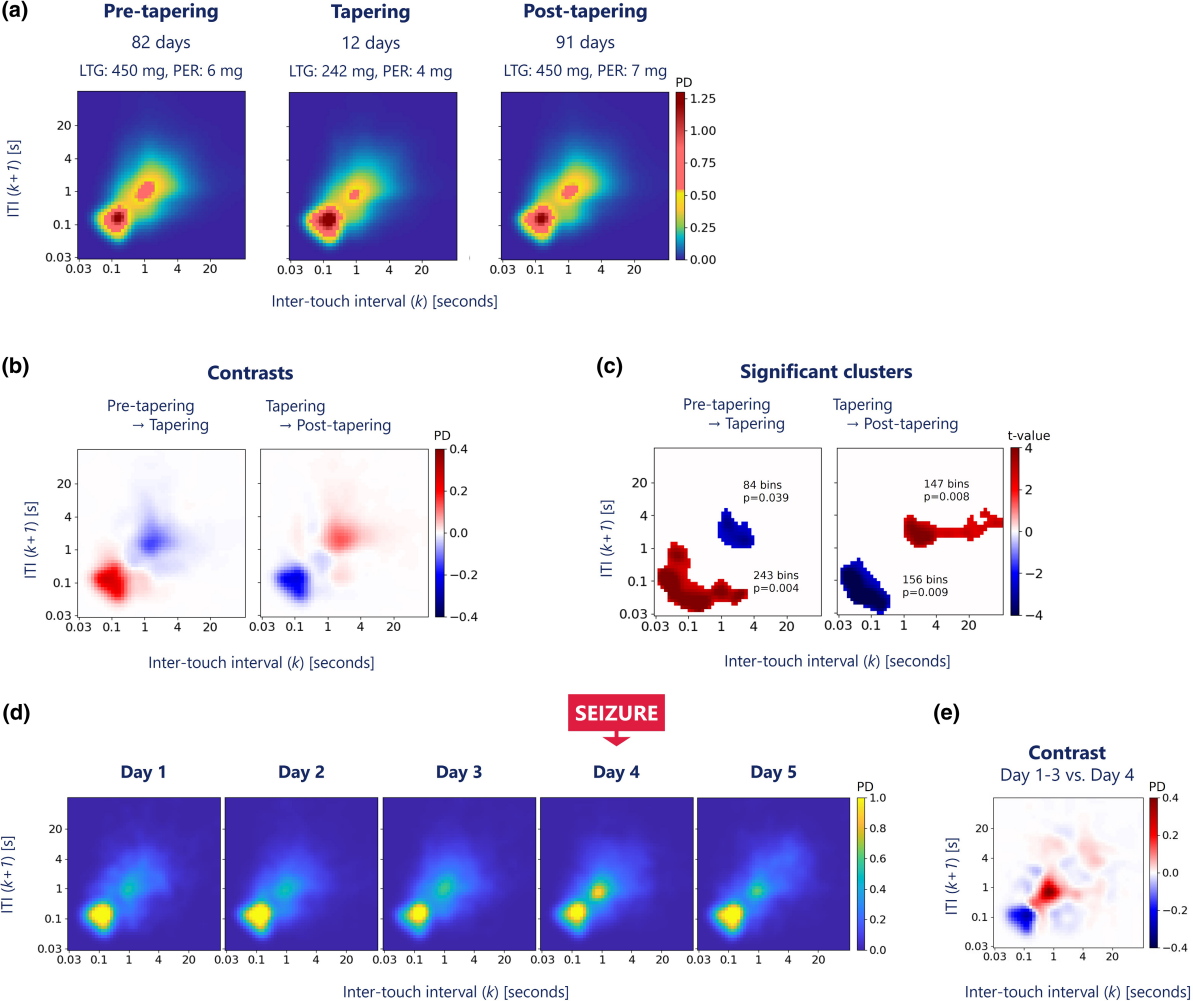
(a) Daily joint interval distributions (JIDs) of inter‐touch intervals averaged for three periods: pre‐tapering, tapering and post‐tapering period. (b) Contrasts between the daily JIDs for both medication transitions (i.e., pre‐tapering → tapering and tapering → post‐tapering) showing an acceleration of next‐interval dynamics during tapering that reversed on medication restart. (c) Results of the cluster‐based permutation testing revealing significant clusters for both medication transitions, which overlapped with the changes displayed in panel B. From pre‐tapering to tapering, the median size of the largest cluster under the null hypothesis was 17 (95% confidence interval (CI) 11 to 27) 2D bins. From tapering to post‐tapering, the median size of the largest cluster under the null hypothesis was 18 (95% CI 11 to 29) 2D bins. *p*‐values reflect the probability of finding equal or greater maximal cluster sizes under the null hypothesis. (d) Daily touchscreen behavioural patterns during the 5‐day admission. Shortly after rise time on Day 4, the man experienced a convulsive seizure. (e) Contrast between the JID of the day with a convulsion and the average JID of the previous 3 days. The graph shows a deceleration of the next‐interval dynamics on the day of the fbTCS. ITI, inter‐touch interval; LTG, lamotrigine; PD, probability density; PER, perampanel.

Comparison of the two medication load transitions indicated an increased probability of fast next‐interval dynamics following tapering, which reversed after medication restart (Figure 2a,b). The permutation‐based clustering test identified significant clusters that overlapped these behavioural changes (Figure 2c).

The probability distributions of the interactions showed marked contrasts between the days with and without focal to bilateral tonic–clonic convulsion, expressing an increased probability of slow next‐interval dynamics on the day of the convulsion compared to the preceding 3 days (Figure 2d,e).

## DISCUSSION

We found marked behavioural changes following a focal to bilateral tonic–clonic convulsion and in response to ASM changes. These changes were recorded with a regular smartphone with negligible additional burden to the user. Touchscreen interactions show an increased probability of slow next‐interval dynamics following a convulsion and increasing ASM load. In contrast, tapering showed the opposite (i.e., an increased likelihood of fast next‐interval dynamics). The pattern is similar to what is seen with increasing chronological age [4, 9].

Cognitive side effects are commonly reported by ASM users and they are a robust predictor of reduced quality of life [1]. Information on these effects is helpful during the titration of ASMs, but an adequate, unobtrusive tracking method is lacking. Seizures may also aggravate cognitive impairments or mood disorders [10], but are similarly challenging to monitor as self‐reported seizure diaries are often inaccurate [11].

This case suggests smartphones may address the need to track medication and seizure‐related behavioural changes. It also underscores the complexity of the fluctuations in subject status that cannot be captured by cross‐sectional assessments such as questionnaires or self‐reports. The neurobehavioural substrates are still largely unexplored, but there is some evidence that touchscreen interactions correlate with conventional assessments of reaction time [12] and reliably estimate biological age [4]. Interestingly, epileptiform discharges coincide with distinct profiles, suggesting that even subtle changes can be monitored [7].

We propose that the increased behavioural ageing in epilepsy is partly explained by ASM exposure and seizure occurrence. ASMs may cause a broad spectrum of cognitive changes, including impaired executive function, attention and reaction time [13]. Our finding of significant behavioural changes following ASM load adjustment is important because the cognitive side effects of the ASMs prescribed to the subject (lamotrigine and perampanel) are relatively unknown but relevant [14, 15]. More pronounced changes in smartphone behaviour could be expected with ASMs associated with marked adverse cognitive effects or with polytherapy [13]. Notably, the net accelerating effect of ASM tapering was significant. However, the period also included the day of the convulsive event, which showed a reverse (i.e., decelerating) effect on smartphone behaviour.

We grouped touchscreen interactions into daily rather than hourly profiles to avoid variability from intermittent smartphone use. While the interaction profile of the day with focal to bilateral tonic–clonic convulsion included a pre‐ and post‐ictal phase, it predominantly reflects the post‐ictal phase as the seizure occurred soon after wake‐up. The post‐ictal state can manifest in a variety of ways, including altered consciousness and cognitive dysfunction [16]. Cognitive impairments may last for hours or even days after a focal to bilateral tonic–clonic convulsion. Our subject's loss of rapid touchscreen interactions may well reflect these post‐ictal signs.

We only examined the effects of the convulsion without evaluating the focal seizures. We anticipated that the cognitive impact of focal seizures, particularly those with retained awareness, is considerably less. Many seizures recorded and a sufficiently long seizure‐free period are necessary to evaluate their effect on smartphone behaviour. We selected a single case to demonstrate the impact of medication withdrawal and the recorded convulsion. Part of the study period took place in the EMU, an unnatural environment that may have influenced our results. Cohort studies in a natural environment are needed to establish our findings' generalisability and ascertain the potential of touchscreen profiles to improve neurobehavioural monitoring in neurological care.

## AUTHOR CONTRIBUTIONS

**Arthur R. van Nieuw Amerongen:** Methodology; formal analysis; investigation; writing – original draft. **Anne Marthe Meppelink:** Writing – review and editing. **Arko Ghosh:** Writing – review and editing; methodology; conceptualization. **Roland D. Thijs:** Writing – review and editing; methodology; conceptualization.

## CONFLICT OF INTEREST STATEMENT

A.G. is a cofounder of QuantActions AG, Zurich, Switzerland, which focus on converting smartphone touchscreen interactions to mental health indicators. Software and data services from QuantActions were used to monitor smartphone activity. A.G. is an inventor on a granted patent that enables smartphone‐based collection of touchscreen interaction timings (US16/315663). R.D.T. reports personal fees from UCB, Theravarance, LivAssured, Novartis, Zogenix, Arvelle, Angelini, Medtronic, and NewLife Wearables.

## ETHICS STATEMENT

The Medical Ethics Committee (Leiden‐Den Haag‐Delft‐P19.088) approved the study.

## PATIENT CONSENT STATEMENT

The subject provided informed consent for participation.

## CLINICAL TRIAL REGISTRATION

This study forms part of a registered clinical trial (https://clinicaltrials.gov/study/NCT04617418).

## Data Availability

The data supporting this study's findings are available from the corresponding author upon reasonable request. The data are not publicly available due to privacy or ethical restrictions. The codes used in this study are available at a Git repository: https://github.com/CODELABCODELIB/Casestudy_epi_SEIN_2023.
